# Supplementary material for: The Oligosaccharide Region of LPS Governs Predation of E. coli by the Bacterivorous Protist, Acanthamoeba castellanii
Source: Microbiol Spectr. 2023 Jan 17;11(1):e02930-22. doi: 10.1128/spectrum.02930-22 (PMC9927288; doi:10.1128/spectrum.02930-22)
Supplement: Supplemental file 1 — Figures S1 and S2. Download spectrum.02930-22-s0001.pdf, PDF file, 0.2 MB [file spectrum.02930-22-s0001.pdf]

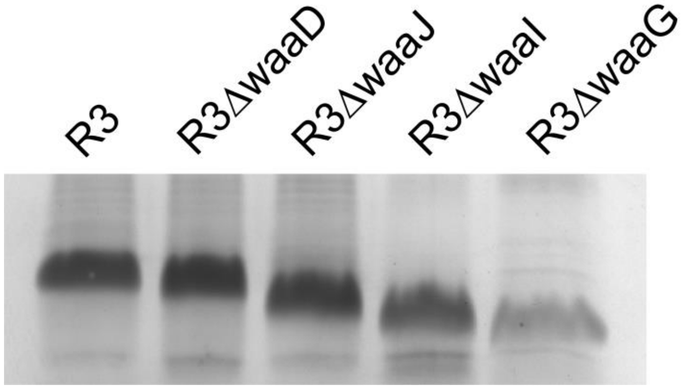

Figure S1: **Extraction and visualization of LPS from R3 and its waa mutants.** LPS of R3 and its mutants: R3ΔwaaD, R3ΔwaaJ, R3ΔwaaI and R3ΔwaaG was extracted using hot phenol. LPS was visualized on 14% SDS-PAGE followed silver staining.

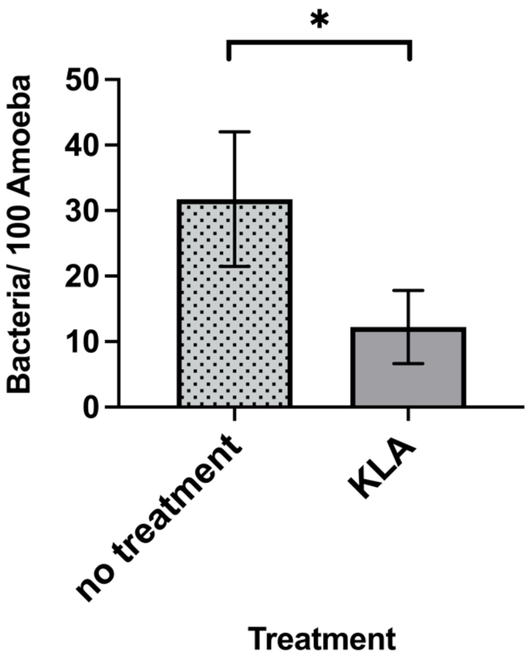

Figure S2: **Effect of Kdo2-Lipid A on *E. coli* recognition and internalization by *Acanthamoeba* in gentamycin protection assay.** *A. castellanii* were separately incubated with 40μg/mL Kdo2-Lipid (KLA) or PAS in 24 well plate for 1 h at 30 °C. *A. castellanii* were then co-cultured with MG1655 for 2 hour at 30 °C. Subsequent to incubation, amoebae cells were washed and 150 µg/ml gentamicin was added. The treated amoeba were then counted, lysed by an addition of 0.2% of sodium dodecyl sulfate, and the lysate was spread on LB agar plates for counting internalized bacteria. Results are normalized to no treatment group. Error bars represent standard deviations from $\geq$3 independent experiment, each has at least two technical replicates. T-test(two-tailed distribution, heteroscedastic) was performed for pairwise comparison. *: $P < 0.05$; NS: not significant $P > 0.05$.
